# TP53 Mutations Unfavorably Impact the Outcomes of Myelofibrosis Patients with or Without Hematopoietic Stem Cell Transplantation: A Single-Center Study and Meta-Analysis

**DOI:** 10.3390/ijms27114729

**Published:** 2026-05-25

**Authors:** Filippo Frioni, Sabrina Giammarco, Sara Ceglie, Silvia Betti, Francesco Ramundo, John Marra, Monica Rossi, Federica Fosso, Gessica Minnella, Elisabetta Metafuni, Federica Sorà, Andrea Bacigalupo, Simona Sica, Elena Rossi, Valerio De Stefano, Patrizia Chiusolo

**Affiliations:** 1 Section of Hematology, Department of Laboratory and Hematological Sciences, Catholic University, 00168 Rome, Italy; 2 Fondazione Policlinico Universitario A. Gemelli IRCCS, 00168 Rome, Italy

**Keywords:** TP53 mutation, Myelofibrosis, hematopoietic stem cell transplantation

## Abstract

TP53 mutation is known to be a poor prognostic factor in many hematologic malignancies, but it is not included in any Myelofibrosis-specific prognostic score. Recently, studies have shown that TP53 mutation may negatively impact the outcomes of patients with Myelofibrosis, including those undergoing hematopoietic stem cell transplantation. In this study, we aimed to analyze the impact of TP53 mutation on the overall survival and leukemia-free survival of patients with chronic-phase Myelofibrosis, regardless of whether they had undergone transplantation. A total of 250 patients were included in the study, of whom 9 (3.2%) harbored a TP53 mutation, including six (3.2%) among non-HSCT patients and three (3.1%) among HSCT patients. In both groups, the presence of TP53 mutation correlated with shorter overall survival, while in non-HSCT patients, it also correlated with lower leukemia-free survival. Moreover, we provide a meta-analysis on the role of TP53 mutation in patients with chronic-phase Myelofibrosis, confirming its negative prognostic impact on overall survival.

## 1. Introduction

Myelofibrosis (MF) is a rare chronic myeloproliferative neoplasm associated with poor survival. It can be primary or secondary, following polycythemia vera (PV) or essential thrombocythemia (ET) [1]. In approximately 20% of cases, MF can progress to acute myeloid leukemia (AML) [1]. Treatment options include anemia-oriented therapies, JAK inhibitors, clinical trials, and allogeneic hematopoietic stem cell transplantation (HSCT), depending on the patient’s prognostic stratification1. High-risk mutations (IDH1, IDH2, SRSF2, EZH2, ASXL1, U2AF1-Q157P) are associated with a worse prognosis [2,3,4,5,6,7]. Only a few prognostic models for MF include TP53 mutation [8,9], and none specifically analyze patients with overt fibrotic-stage MF.

## 2. Results

The cohort consisted of 251 patients (characteristics shown in Table 1 and Supplementary Table S1), of whom 96 high-risk eligible patients underwent HSCT.

Of the nine TP53-mutated patients (3.5%), six (3.8%) belonged to the non-HSCT cohort and three (3.1%) to the HSCT cohort. Notably, all six TP53-mutated patients in the non-HSCT cohort harbored single-hit TP53 mutations, and none of the TP53-mutated patients had chromosome 1 translocation/amplification. The characteristics of the nine TP53-mutated patients are summarized in Supplementary Table S3.

### 2.1. Non-HSCT Patients

#### 2.1.1. Leukemia-Free Survival

**Figure 1:**
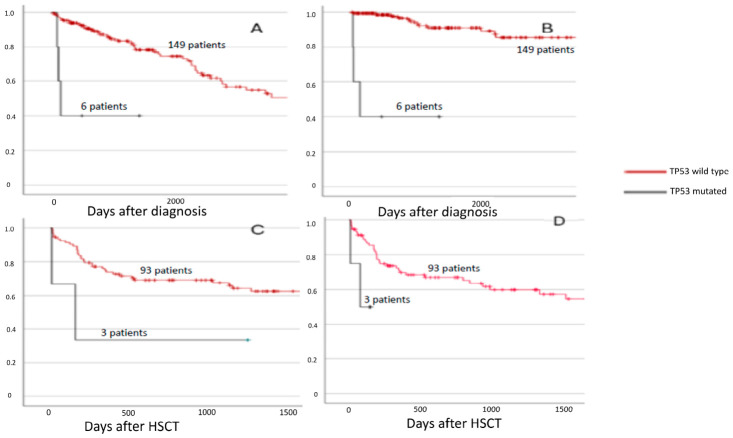
(**A**). Kaplan–Meier analysis of OS in non-HSCT patients (**B**). Kaplan–Meier analysis of LFS in non-HSCT patients (**C**). Kaplan–Meier analysis of OS in HSCT patients (**D**). Kaplan–Meier analysis of RFS in HSCT patients.

Kaplan–Meier analysis showed that LFS was shorter in TP53-mutated patients (*p* < 0.001, Figure 1), with survival estimates reported with 95% confidence intervals.

LFS was significantly associated with EZH2, SRSF2, single-hit TP53, SETBP1, DNMT3A, and IKZF1 mutations. Anemia, peripheral blood myeloblasts ≥2%, constitutional symptoms, and grade 3 bone marrow fibrosis were also associated with shorter LFS (Supplementary Table S2). Multivariable analysis confirmed that TP53 retained an independent prognostic role.

#### 2.1.2. Overall Survival

OS in non-HSCT patients was lower in TP53-mutated patients according to Kaplan–Meier analysis (*p* < 0.001, Figure 1), with survival estimates reported with 95% confidence intervals.

Mutations in EZH2, IDH1, SRSF2, CBL, TP53, and SETBP1 were associated with shorter OS. In addition, unfavorable karyotype, anemia, leukocytosis, thrombocytopenia, peripheral blood myeloblasts ≥2%, constitutional symptoms, and grade 3 bone marrow fibrosis were also associated with worse OS. In contrast, secondary MF and previous treatment with Ruxolitinib were associated with better OS. In multivariable analysis, TP53 mutation (*p* = 0.002) retained statistical significance.

Detailed results of the multivariable analyses for LFS and OS are reported in Supplementary Table S2.

### 2.2. HSCT Patients

#### 2.2.1. Overall Survival

We first examined, in HSCT patients, the eight variables that significantly impacted OS in non-HSCT patients at multivariable analysis: EZH2, SRSF2, and TP53 mutations; unfavorable karyotype; peripheral blood myeloblasts ≥2%; constitutional symptoms; grade 3 bone marrow fibrosis; and previous treatment with Ruxolitinib.

Only TP53 (*p* = 0.06) and SRSF2 (*p* = 0.065) showed a strong association with lower OS in univariable analysis. In multivariable analysis, TP53 (*p* = 0.043) and SRSF2 (*p* = 0.0499) were independently and significantly associated with worse OS (Supplementary Table S2).

Kaplan–Meier analysis confirmed that OS was shorter in TP53-mutated patients (*p* = 0.06, Figure 1), with survival estimates reported with 95% confidence intervals.

We then reanalyzed all variables, including transplant-specific ones. Univariable analysis revealed that SRSF2 mutation, TP53 mutation, ETV6 mutation, age <57 years, thrombocytopenia, and infusion of fewer than 5 × 10^6^/kg CD34+ cells significantly impacted OS. In multivariable analysis, only TP53 mutation (*p* = 0.03), ETV6 mutation (*p* = 0.04), and the number of infused CD34+ cells (*p* = 0.003) remained statistically significant.

#### 2.2.2. Relapse-Free Survival

RFS was shorter in TP53-mutated patients after transplantation (Figure 1).

IDH1 mutation (*p* = 0.02), TP53 mutation (*p* = 0.01), and age >57 years (*p* = 0.03) were associated with lower RFS. However, in multivariable analysis, only TP53 mutation retained statistical significance (*p* = 0.02).

### 2.3. Meta-Analysis

We found that TP53 and SRSF2 mutations were the only mutations associated with shorter OS in both non-HSCT and HSCT patients. Therefore, we conducted a meta-analysis evaluating the impact of these mutations on OS in patients with MF.

TP53 mutation was associated with shorter OS both when considering all studies (*p* < 0.001, risk ratio = 2.63) and when excluding studies on HSCT patients (*p* < 0.001, risk ratio = 5.93). Additionally, SRSF2 mutation was associated with lower OS when considering all studies (*p* = 0.06, risk ratio = 1.3); however, a meta-analysis excluding studies on HSCT patients was not feasible due to the limited number of available studies.

**Figure 2:**
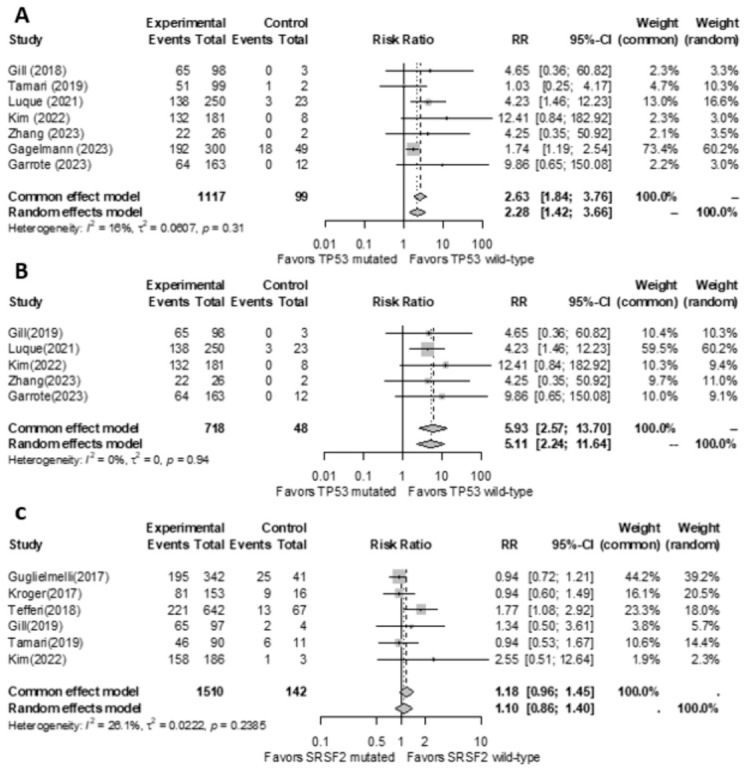
(**A**). Meta-analysis of the impact of the TP53 mutation on the overall survival in HSCT and non-HSCT patients with Myelofibrosis. (**B**). Meta-analysis of the impact of the TP53 mutation on the overall survival in selected non-HSCT patients with Myelofibrosis. (**C**). Meta-analysis of the impact of the SRSF2 mutation on the overall survival in HSCT and non-HSCT patients with Myelofibrosis. The cited references are [10,11,12,13,14,15,16,17,18,19].

Forest plots for OS are shown in Figure 2. A larger meta-analysis including studies based on the 2008 WHO classification, which also confirmed the negative impact of TP53 mutation, is presented in the Supplementary Materials (Supplementary Figure S7).

## 3. Discussion

TP53 mutation is not currently considered in many prognostic models for patients with overt fibrotic-phase MF. The first report on TP53-mutated patients with MF concluded that TP53 mutation did not impact overall survival [20], and TP53 was not included among the hotspot genes for myeloid malignancies analyzed in the MIPSS70+ score [21]. Moreover, the rarity of TP53 mutations (2–4% of all MF cases) makes their prognostic impact difficult to assess [22]. However, recent studies have shed light on the role of TP53 mutations in patients with MF [12,15]. In particular, a recent multicenter study identified a very high-risk subgroup among multi-hit TP53-mutated HSCT patients [15].

We identified eight independent variables with a prognostic impact on OS in non-HSCT patients: SRSF2 mutation, EZH2 mutation, unfavorable karyotype, constitutional symptoms, grade 3 bone marrow fibrosis, previous treatment with Ruxolitinib, peripheral blood myeloblasts ≥2%, and TP53 mutation. We further analyzed the role of these variables in HSCT patients and found that only SRSF2 and TP53 mutations retained a prognostic role.

Furthermore, we found that EZH2 mutation, DNMT3A mutation, SETBP1 mutation, constitutional symptoms, peripheral blood myeloblasts ≥2%, and TP53 mutation were independent predictors of leukemic transformation. Interestingly, all six TP53-mutated patients in our non-HSCT cohort harbored single-hit TP53 mutations, enabling us to assess the prognostic significance of single-hit TP53 mutation in chronic-phase MF patients not undergoing HSCT, despite its rarity. Moreover, in our study DNMT3A, EZH2, and SETBP1 mutations were associated with an increased risk of leukemic transformation, suggesting that, although further studies specifically addressing this aspect are needed, these mutations should be considered in the prognostic stratification of patients.

We also examined a subset of 96 patients who underwent allogeneic HSCT. TP53 mutation, ETV6 mutation, and the number of infused CD34+ stem cells were associated with lower OS in multivariable analysis. We observed that the lower OS in TP53-mutated patients was mainly driven by relapse following HSCT.

Our study also provides the first meta-analysis evaluating the impact of TP53 mutation on OS in patients with chronic-phase MF, as well as the second meta-analysis regarding SRSF2 mutation in the same patient setting (Figure 2). Both TP53 and SRSF2 mutations showed a detrimental prognostic role in patients with overt fibrotic-stage MF.

In conclusion, our study highlights the detrimental effect of TP53 mutation in both non-HSCT patients and those undergoing allogeneic HSCT for MF, as previously shown by Gagelmann et al. [15] in multi-hit TP53-mutated transplanted patients, and more recently by Loscocco et al. [23] and Hernandez-Sanchez et al. [24]. These latter two studies analyzed the prognostic contribution of non-driver gene mutations, including TP53 mutations, in both prefibrotic and overt fibrotic-stage MF and were therefore not included in the meta-analysis. Our study differs from that of Loscocco et al. [23] in that we exclusively analyzed patients with overt fibrotic-stage MF, excluding patients with prefibrotic MF. Moreover, unlike the study by Gagelmann et al. [15], which highlighted the detrimental prognostic role of multi-hit TP53 mutations in transplanted patients, the vast majority of TP53-mutated patients in our cohort harbored single-hit TP53 mutations. In addition, our study included both transplanted and non-transplanted patients, allowing us to evaluate the prognostic impact of TP53 mutations across both clinical settings.

Although our study has some limitations, such as its retrospective design and the low number of TP53-mutated patients, it is important to note that, due to the rarity of the mutation, our sample size is comparable to that of most published case series [11,20,22].

## 4. Methods

This single-center study involved 251 adult patients with primary or secondary MF, as defined by the WHO and the ICC of Myeloid Neoplasms for primary MF [25,26] and by the IWG-MRT criteria for secondary MF [27]. Patients with pre-fibrotic stage MF, accelerated-phase MF, and post-MF AML were excluded. Table 1 summarizes the characteristics of the 251 patients. The local Ethics Committee approved this study (Protocol ID 3360).

Next-generation sequencing (NGS) analysis was performed using the Illumina^®^ MiniSeq instrument on bone marrow samples collected at diagnosis. Detailed information on the custom gene panel is provided in the Supplementary Materials.

We first analyzed the overall survival (OS) of TP53-mutated and TP53 wild-type patients in both the non-HSCT and HSCT populations, as well as leukemia-free survival (LFS) in non-HSCT patients and relapse-free survival (RFS) in HSCT patients, using the Kaplan–Meier estimation. We then conducted an analysis to determine how various clinical, biochemical, histological, cytogenetic, and molecular factors at diagnosis (as outlined in Supplementary Table S2) affected OS, LFS, and RFS. Unfavorable cytogenetics were defined according to MIPSS70+ [21]. Single-hit and multi-hit TP53 mutations were defined according to Arber et al. [26].

For non-HSCT patients, OS and LFS were calculated from diagnosis until death or AML development, respectively. For HSCT patients, RFS was calculated from the time of transplantation to the date of relapse. Factors associated with shortened OS in non-HSCT patients were also analyzed in HSCT patients. Finally, we analyzed the impact of each variable on OS and RFS in HSCT patients only, in addition to transplant-specific variables such as the HSCT-specific Comorbidity Index (HCT-CI), donor type, stem cell source, number of CD34+ and CD3+ cells infused with the graft, conditioning regimen, graft-versus-host disease (GvHD) prophylaxis, and GvHD occurrence.

Univariable and multivariable analyses were performed using the Cox proportional hazards regression model. In the multivariable analysis, only variables with a *p*-value < 0.1 in the univariable analysis were included. Statistical analyses were performed using R software version 4.2.1.

Meta-analyses were conducted to determine whether TP53 and SRSF2 mutations have an impact on the overall survival of patients with myelofibrosis, regardless of whether they were treated with allogeneic HSCT or not. The meta-analyses were performed following PRISMA guidelines [28] and utilizing the “meta” package in R software (version 4.21, “meta” package version 6.5) [29]. In view of the high likelihood of variations among study samples, the random effects model was used to assess relative risk, 95% confidence intervals, and *p*-values [30]. Heterogeneity among the studies was measured using the I2 statistic [31]. Subgroup meta-analyses were performed within cohorts of patients who did not receive an allogeneic HSCT, while subgroup meta-analyses within cohorts of patients treated with allogeneic HSCT could not be conducted due to the insufficient number of study samples. A *p*-value below 0.05 was regarded as statistically significant. Detailed information regarding the literature search, article selection strategy, and risk of bias assessment (performed according to McGuinness et al. [32]) is available in the Supplementary Materials (Supplementary Materials and Supplementary Figures S1–S6).

## 5. Conclusions

Integrating TP53 mutation in the prognostic evaluation of MF should be appropriate in selecting the most accurate patient-tailored treatment strategy.

## Figures and Tables

**Table 1:** Characteristics of patients.

Variable	Non-HSCT Patients (n = 155)	HSCT Patients (n= 96)	*p*
Sex	F: 83	F: 43	0.16
	M: 72	M: 53	
Diagnosis	Post-PV MF: 34	Post-PV MF: 19	0.62
	Post-TE MF: 46	Post-TE MF: 36	
	MFI: 75	MFI: 41	
Driver mutation	JAK2: 106	JAK2: 68	0.91
	CALR: 26	CALR: 15	
	MPL: 10	MPL: 7	
	TN: 13	TN: 6	
Bone marrow fibrosis (grade at the onset)	Grade 1: 49	Grade 1: 31	0.28
	Grade 2: 40	Grade 2: 34	
	Grade 3: 31	Grade 3: 31	
	Not evaluable: 35		
Thrombotic events	Yes: 27	Yes: 20	0.51
	No: 128	No: 76	
Median age (years) (range 29–91)	61	54	<0.001
Previous treatment with Ruxolitinib	Yes: 66	Yes: 70	<0.001
	No: 89	No: 26	
Splenomegaly	Yes: 114	Yes: 91	<0.001
	No: 41	No: 5	
Unfavourable Cytogenetic	Yes: 21	Yes: 36	<0.001
	No: 134	No: 60	
Detrimental Mutations	Yes: 55	Yes: 67	<0.001
	No: 100	No: 32	
Other non-detrimental mutations	Yes: 58	Yes: 60	<0.001
	No: 97	No: 36	
Conditioning regimen		Reduced intensity conditioning: 94	
		Myeloablative conditioning: 2	
Donor		MUD 8/8: 38	
		UD: 12	
		HLA-identical sibling: 23	
		Haploidentical: 22	
		Cord blood: 1	
GvHD prophylaxys		ptCy: 81	
		Other: 15	
HSC source		Peripheral blood: 74	
		Bone marrow: 21	
		Cord blood: 1	
GvHD grade ≥ 2		18	

## Data Availability

For original data, please contact via e-mail: patrizia.chiusolo@unicatt.it.

## Supplementary Materials

The following supporting information can be downloaded at: https://www.mdpi.com/article/10.3390/ijms27114729/s1. Reference [33] is cite in supplementary materials.
