# Supplementary material for: TP53 Mutations Unfavorably Impact the Outcomes of Myelofibrosis Patients with or Without Hematopoietic Stem Cell Transplantation: A Single-Center Study and Meta-Analysis
Source: Int J Mol Sci. 2026 May 25;27(11):4729. doi: 10.3390/ijms27114729 (PMC13256192; doi:10.3390/ijms27114729)
Supplement: Supplementary file 1 [file ijms-27-04729-s001.zip › ijms-4303381-supplementary.pdf]

TP53 mutations unfavorably impact the outcomes of myelofibrosis patients with or without hematopoietic stem cell transplantation: a single-center study and meta-analysis

Filippo Frioni[1 #], Sabrina Giammarco[2 #], Sara Ceglie[2], Silvia Betti[2], Francesco Ramundo[1], John Marra[1], Monica Rossi[2], Federica Fosso[2], Gessica Minnella[2], Elisabetta Metafuni[2], Federica Sorà[1,2], Andrea Bacigalupo[2], Simona Sica[1,2], Elena Rossi[1, 2], Valerio De Stefano[1, 2 §], Patrizia Chiusolo [1,2 §]

[1]Section of Hematology, Department of Radiological and Hematological Sciences, Catholic University, Rome, Italy

[2]Fondazione Policlinico Universitario A. Gemelli IRCCS, Rome, Italy

Statement of equal author contribution

# FFr and SG contributed equally to the study.

§ VDS and PC contributed equally to the study.

Supplemental Material: Custom gene panel

Custom gene panel included the following genes: JAK2, CALR, MPL, RUNX1, TP53, EZH2, CUX1, REEP5, IKZF1, TET2, KIT, CBL, SF3B1, SRSF2, DNMT3A, ASXL1, IDH1, U2AF1, IDH2, CSF3R, PDL5, CBLB, NRAS, KRAS, ZRSR2, WT1, ETV6.

Supplemental Material: Data Sources and Searches

We used the following search strings for Pubmed, Embase, Scopus

1) TP53 AND Myelofibrosis
2) TP53 AND Primary Myelofibrosis
3) TP53 AND Secondary Myelofibrosis
4) SRSF2 AND Myelofibrosis
5) SRSF2 AND Primary Myelofibrosis
6) SRSF2 AND Secondary Myelofibrosis

The search was made until March, 20th, 2023

The meta-analyses were performed following PRISMA guidelines (Moher et al. Preferred reporting items for systematic reviews and meta-analyses: the PRISMA statement. J Clin Epidemiol. 2009 Oct;62(10):1006-12.) and utilizing the "meta" package in R software version 4.21, "meta" package version 6.5 (Balduzzi et al. How to perform a meta-analysis with R: a practical tutorial. Evid Based Ment Health. 2019 Nov;22(4):153-160). Since no article published before 2016 distinguished between pre-fibrotic and overt fibrotic stage MF, we considered for the meta-analysis only studies published after 2016 WHO classification of Myeloid Neoplasms, still excluding those specifying in the "Methods" section that the diagnosis was made according to 2008 WHO classification. As regards of articles about both pre-fibrotic and overt fibrotic stage MF, only patients

diagnosed with overt fibrotic stage MF were considered in the meta-analysis. Subgroup meta-analyses were performed within cohorts of patients who did not receive an allogeneic HSCT, while subgroup meta-analyses within cohorts of patients treated with allogeneic HSCT could not be conducted due to the insufficient number of study samples. We then performed a larger meta-analysis taking into account also articles published before 2016 WHO classification. In accordance with the heterogeneity among study samples, the common effect model (in case of low heterogeneity) or the random effect model (in case of high heterogeneity) was used to assess relative risk, 95% confidence intervals, and p-values (Borenstein et al. A basic introduction to fixed-effect and random-effects models for meta-analysis. Res Synth Methods. 2010 Apr;1(2):97-111). Heterogeneity among the studies was measured using the I2 statistic (Higgins JP, Thompson SG, Deeks JJ, Altman DG. Measuring inconsistency in meta-analyses. BMJ. 2003 Sep 6;327(7414):557-60). A p-value below 0.05 was regarded as statistically significant. In TP53 mutation meta-analysis, the common effect model was preferred due to the low heterogeneity of the studies, while in SRSF2 meta-analysis the random effect model was preferred.

Supplemental Material: Meta-analysis references

1. Gill H, Ip HW, Yim R, Tang WF, Pang HH, Lee P, et al. Next-generation sequencing with a 54-gene panel identified unique mutational profile and prognostic markers in Chinese patients with myelofibrosis. Ann Hematol. 2019 Apr;98(4):869-879.
2. Tamari R, Rapaport F, Zhang N, McNamara C, Kuykendall A, Sallman DA, et al. Impact of High-Molecular-Risk Mutations on Transplantation Outcomes in Patients with Myelofibrosis. Biol Blood Marrow Transplant. 2019 Jun;25(6):1142-1151.
3. Courtier F, Garnier S, Carbuccia N, Guille A, Adélaide J, Chaffanet M, et al. Targeted molecular characterization shows differences between primary and secondary myelofibrosis. Genes Chromosomes Cancer. 2020 Jan;59(1):30-39.
4. Luque Paz D, Riou J, Verger E, Cassinat B, Chauveau A, Ianotto JC, et al. Genomic analysis of primary and secondary myelofibrosis redefines the prognostic impact of ASXL1 mutations: a FIM study. Blood Adv. 2021 Mar 9;5(5):1442-1451.
5. Kim TY, Kwag D, Lee JH, Lee J, Min GJ, Park SS, et al. Clinical Features, Gene Alterations, and Outcomes in Prefibrotic and Overt Primary and Secondary Myelofibrotic Patients. Cancers (Basel). 2022 Sep 16;14(18):4485.
6. Zhang L, Ye X, Luo S, Xu X, Wang S, Jin K, et al. Clinical features and next-generation sequencing landscape of essential thrombocythemia, prefibrotic primary myelofibrosis, and overt fibrotic primary myelofibrosis: a Chinese monocentric retrospective study. J Cancer Res Clin Oncol. 2023 Jun;149(6):2383-2392.
7. Gagelmann N, Badbaran A, Salit RB, Schroeder T, Gurnari C, Pagliuca S, et al. Impact of TP53 on outcome of patients with myelofibrosis undergoing hematopoietic stem cell transplantation. Blood. 2023 Jun 8;141(23):2901-2911.
8. Garrote M, López-Guerra M, Arellano-Rodrigo E, Rozman M, Carbonell S, Guijarro F, et al. Clinical Characteristics and Outcomes of Patients with Primary and Secondary Myelofibrosis According to the Genomic Classification Using Targeted Next-Generation Sequencing. Cancers (Basel). 2023 Jul 31;15(15):3904
9. Guglielmelli P, Pacilli A, Rotunno G, Rumi E, Rosti V, Delaini F, et al. Presentation and outcome of patients with 2016 WHO diagnosis of prefibrotic and overt primary myelofibrosis. Blood. 2017 Jun 15;129(24):3227-3236.
10. Kröger N, Panagiota V, Badbaran A, Zabelina T, Triviai I, Araujo Cruz MM, et al. Impact of Molecular Genetics on Outcome in Myelofibrosis Patients after Allogeneic Stem Cell Transplantation. Biol Blood Marrow Transplant. 2017 Jul;23(7):1095-1101.
11. Tefferi A, Guglielmelli P, Nicolosi M, Mannelli F, Mudireddy M, Bartalucci N, et al. GIPSS: genetically inspired prognostic scoring system for primary myelofibrosis. Leukemia. 2018 Jul;32(7):1631-1642.

Supplemental Figure S1

TP53 Meta-analysis, decision Algorithm

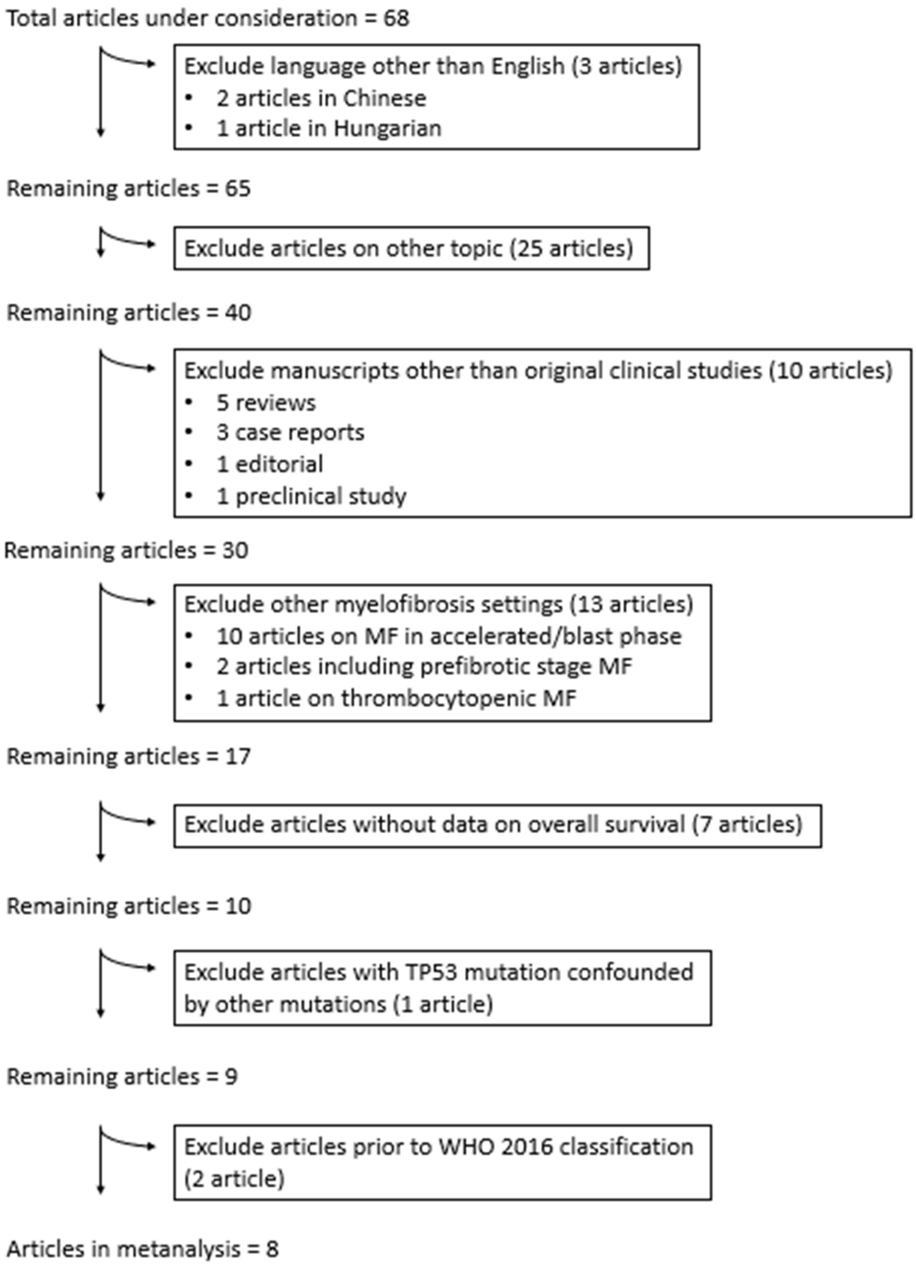

Supplemental Figure S2

SRSF2 Meta-analysis, decision Algorithm

Total articles under consideration = 108

Exclude language other than English (6 articles)
- 5 articles in Chinese
- 1 article in Japanese

Remaining articles = 102

Exclude articles on other topics (40 articles)

Remaining articles = 62

Exclude articles on pediatric cases (1 article)

Remaining articles = 61

Exclude manuscripts other than original clinical studies (26 articles)
- 22 reviews
- 4 case reports

Remaining articles = 35

Exclude other myelofibrosis settings (8 articles)
- 7 articles on MF in accelerated/blast phase
- 1 article on prefibrotic stage MF

Remaining articles = 27

Exclude articles without data on overall survival (7 articles)

Remaining articles = 20

Exclude articles with SRSF2 mutation confounded by other mutations (2 article)

Remaining articles = 18

Exclude articles prior to WHO 2016 classification (10 articles)

Remaining articles = 8

Exclude articles with overlapping cohorts (2 articles)

Articles in metanalysis = 6

Supplemental Figure S3

TP53 Meta-analysis: risk of bias assessment, summary plot

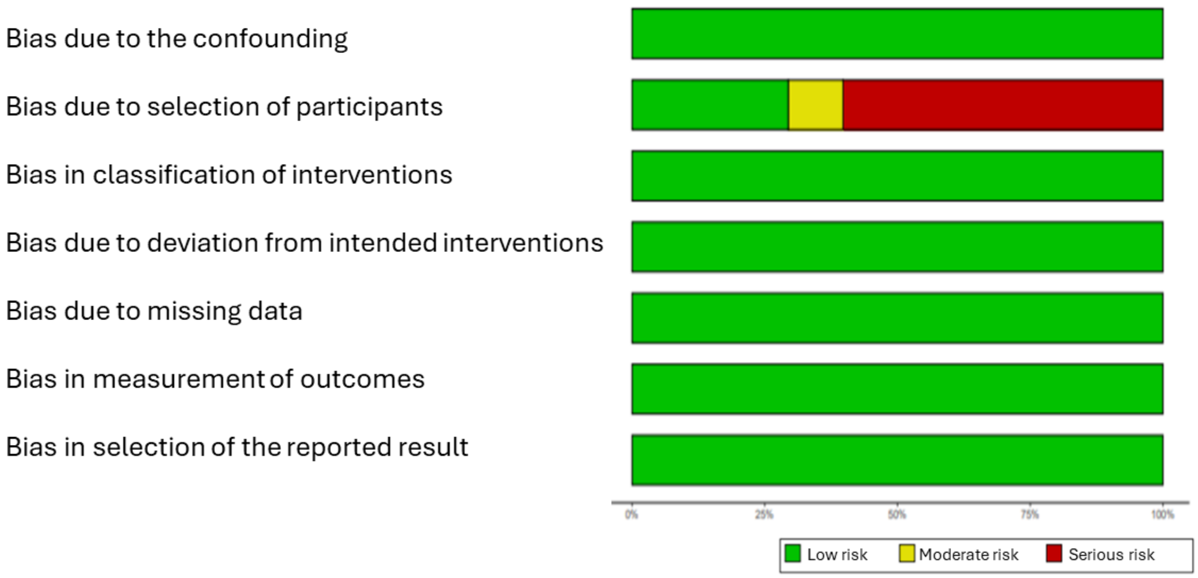

Supplemental Figure S4

TP53 Meta-analysis: risk of bias assessment, traffic light plot

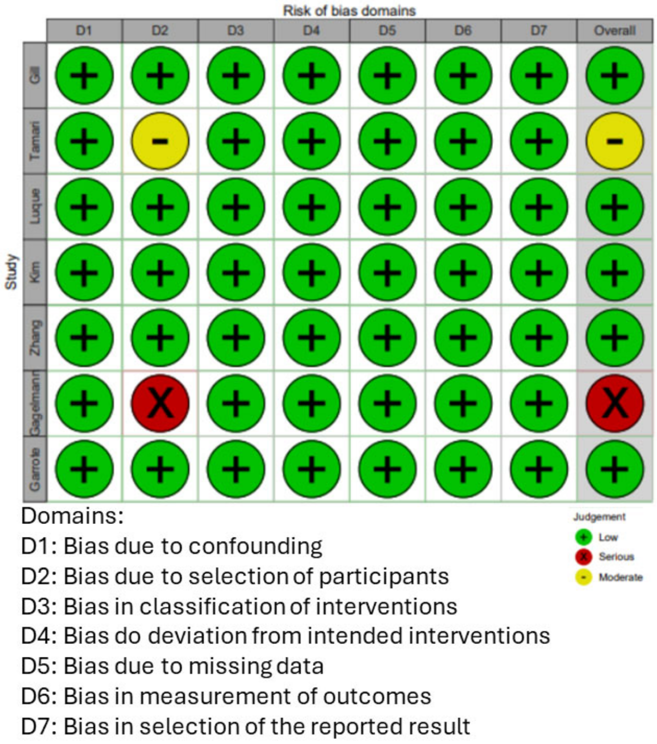

Supplemental Figure S5

SRSF2 Meta-analysis: risk of bias assessment, summary plot

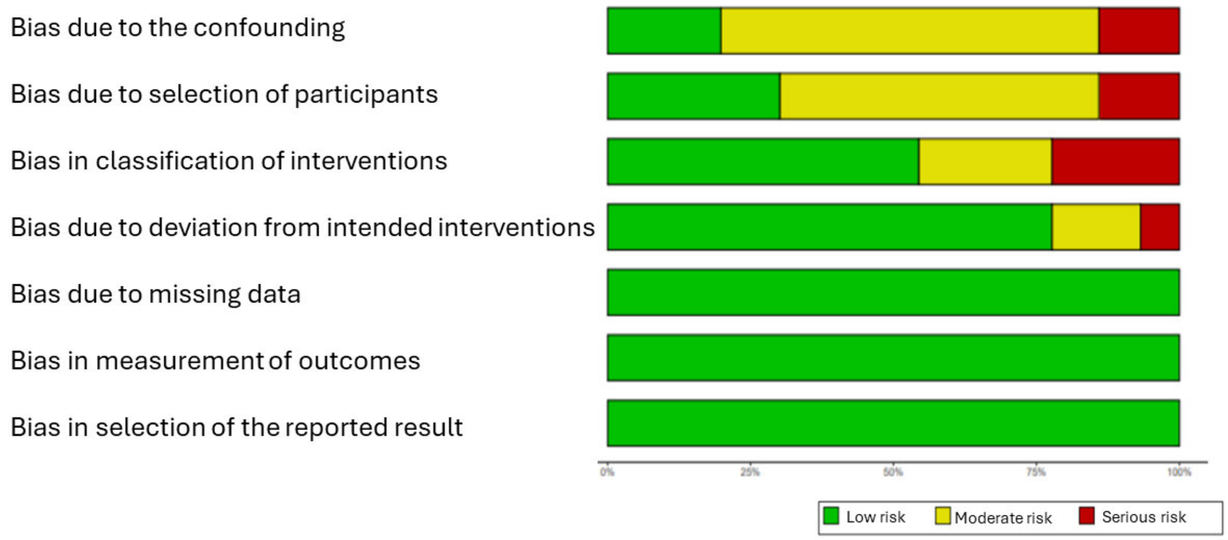

Supplemental Figure S6

SRSF2 Meta-analysis: risk of bias assessment, traffic light plot

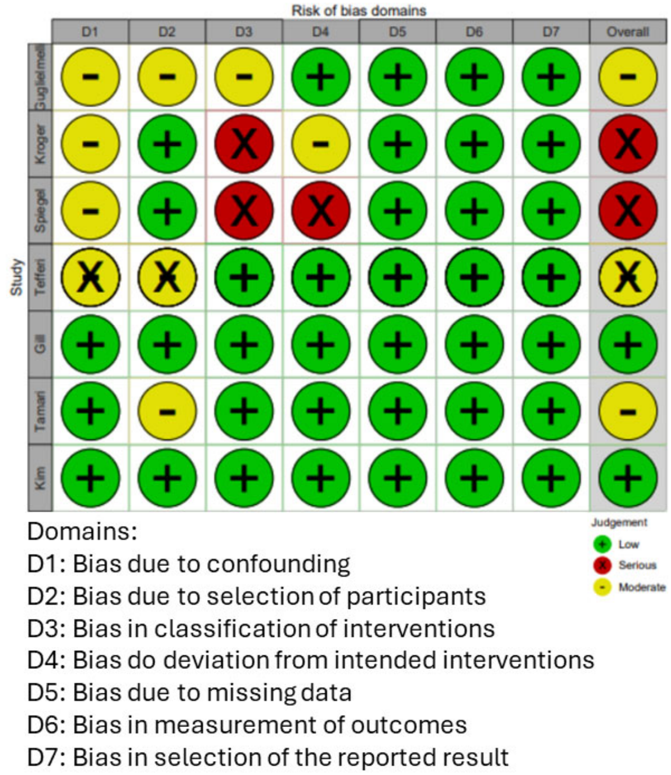

Supplemental Figure S7

TP53 Meta-analysis, including also articles based on 2008 WHO classification

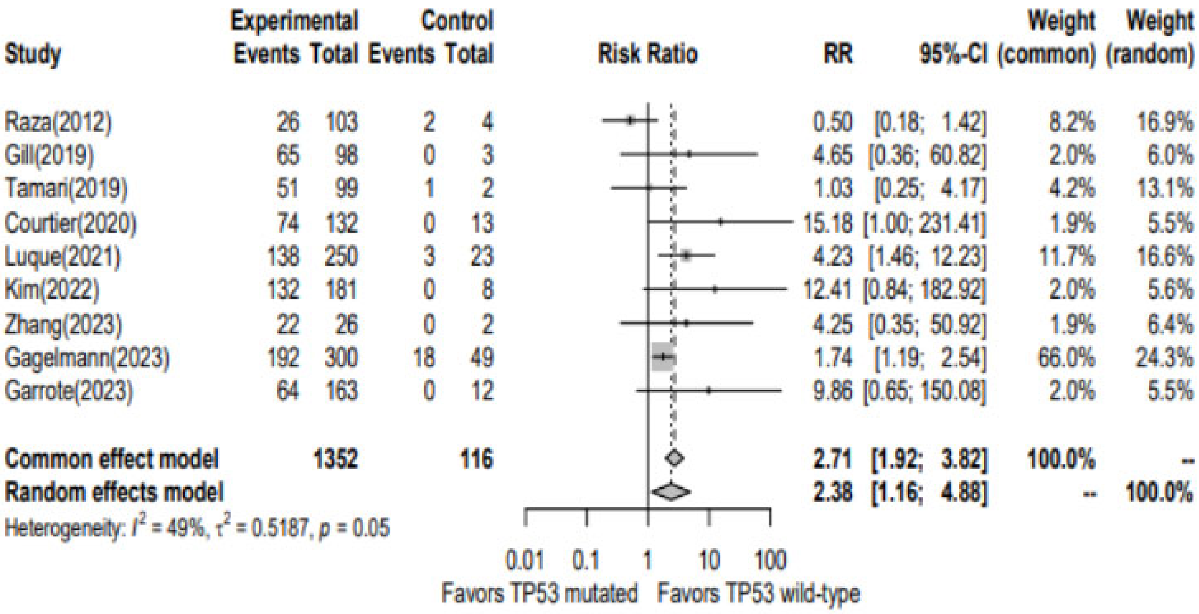

Supplemental Table S1

Non-driver mutations among the whole cohort

Mutation	Number of mutated (%)
ASXL1	78 (31)
EZH2	33 (13)
IDH	20 (8)
SRSF2	22 (9)
U2AF1	21 (8)
SF3B1	26 (10)
TET2	83 (33)
KIT	18 (7)
RUNX1	12 (5)
TP53	9 (3.6)
SETBP1	8 (3)
DNMT3A	18 (7)
CBL	13 (5)
ZRSR2	16 (6)
CUX1	6 (2)
ETV6	3 (1)
IKZF1	3 (1)
CSF3R	2 (1)
RAS	12 (5)

Supplemental Table S2

Univariable and multivariable analysis on overall survival of non-HSCT and HSCT patients (upper panel) and leukemia-free survival (lower panel) of non-HSCT patients. Bold characters indicate statistically significant values.

	**Non-HSCT patients**				**HSCT patients**			
**Variable**	**Univariable analysis**	**Hazard Ratio (CI)**	**Multivariable analysis**	**Hazard Ratio (CI)**	**Univariable analysis**	**Hazard Ratio (CI)**	**Multivariable analysis**	**Hazard Ratio (CI)**
**EZH2**	**p 0.0714**	**2.1 (0.9-4)**	**p 0.01**	**3.9 (1.2-12)**	p 0.4			
**IDH1**	**p 0.003**	**24 (2-201)**	p 0.12					
**SRSF2**	**p <0.001**	**4.18 (1.89-9.2)**	**p 0.03**	**3.7 (1-12.6)**	**p 0.0654**	**2.4 (0.94-6.4)**	**p 0.0499**	**2.6 (1.1-69)**
**CBL**	**p 0.005**	**3.7 (1.4–9.5)**	p 0.2					
**TP53**	**p 0.003**	**6.22 (1.8–20.9)**	**p 0.002**	**18 (2.8-39)**	**p 0.0609**	**3.9 (0.93-16.7)**	**p 0.0432**	**4.5 (1.1-19)**
**SETBP1**	**p <0.001**	**10 (3-34.8)**	p 0.058					
ZRSR2	p 0.1							
RUNX1	p 0.6							
ASXL1	p 0.375							
IDH2	p 0.545							
U2AF1	p 0.327							
TET2	p 0.5							
DNMT3A	p 0.647							
SF3B1	p 0.5							
KIT	p 0.258							
CUX1	p 0.597							
CALR1+ MF	p 0.1							
Triple negative	p 0.159							
**Unfavourable karyotype**	**p <0.001**	**4.5 (1.9–10.5)**	**p 0.01**	**3.8 (1.2-11.5)**	p 0.25			
**Secondary MF**	**p 0.03**	**0.53 (0.3–0.94)**	p 0.2					
**Anemia**	**p <0.001**	**4.2 (2.1-8.3)**	p 0.17					
**Leukocytosis**	**p 0.055**	**2.06 (1.1-13.2)**	p 0.3					
**Thrombocytopenia**	**p 0.0551**	**3.3 (0.9-11)**	p 0.7					
**Peripheral Blood myeloblasts ≥2%**	**p 0.0769**	**2.08 (0.9-4.6)**	**p 0.02**	**4 (1.1-13-8)**	P 0.3			
**Constitutional symptoms**	**p <0.001**	**2.63 (1.5–4.5)**	**p 0.002**	**3.1 (1.4-6.8)**	p 0.5			
**Grade 3 fibrosis**	**p <0.001**	**3.1 (1.6–5.7)**	**p 0.02**	**2.3 (1.1-4.9)**	p 0.91			
Gender	p 0.438							
Age	p 0.85							
LDH	p 0.607							
Splenomegaly	p 0.649							
Cardiovascular risk factors	p 0.481							
**Treatment with Ruxolitinib**	**P 0.005**	**0.4 (0.25–0.79)**	**p 0.001**	**0.2 (0.1-0.6)**	p 0.6			
Cytoreductive therapy	p 0.528							

**Variable**	**Univariable analysis**	**Hazard Ratio (CI)**	**Multivariable analysis**	**Hazard Ratio (CI)**
**EZH2**	**p 0.003**	**5.7 (1.8-18.1)**	**p 0.016**	**23 (1-310)**
**SRSF2**	**p 0.029**	**4.7 (1.3-17)**	p 0.13	
**TP53**	**P<0.001**	**20.2 (5.3–76.8)**	**p<0.001**	**156 (91-267)**
**SETBP1**	**p <0.001**	**9.9 (1.2-79)**	**p 0.02**	**24 (1.4-39.8)**
**DNMT3A**	p 0.06	**3.3 (1-13)**	**p 0.001**	**19 (3-113)**
**IKZF1**	**p 0.003**	**26 (3.1-229)**	p 0.1	
**Anemia**	**p 0.02**	**4.8 (2.1-22)**	p 0.3	
**Peripheral Blood myeloblasts ≥2%**	**p 0.02**	**4.3 (1.3-13)**	**p 0.02**	**13 (1.5-126)**
**Constitutional symptoms**	**p 0.009**	**3.3 (1.2–8.9)**	**p 0.03**	**5.4 (1.1-26)**
**Grade 3 fibrosis**	**p 0.01**	**4.8 (1.3–17)**	p 0.12	

Supplemental Table S3

Clinical and molecular characteristics of the TP53-mutated patients

Median age (range)	60 (49-80)
M/F	6/3
Diagnosis	Primary MF: 3 patients<br>Secondary MF: 6 patients<br>- Post-ET MF: 5 patients<br>- Post-PV MF: 1 patient
Driver mutation	JAK2: 6 patients<br>CALR: 1 patient<br>MPL: 1 patient<br>Triple-negative: 1 patient
Cytogenetic	Favorable: 7 patients<br>Unfavorable: 2 patients
TP53 mutations	Single-hit: 8 patients<br>Multi-hit: 1 patient
Median variant allele frequency (range)	21% (5-84)
